# Advances in immune checkpoint-based immunotherapies for multiple sclerosis: rationale and practice

**DOI:** 10.1186/s12964-023-01289-9

**Published:** 2023-11-09

**Authors:** Amin Daei Sorkhabi, Erfan Komijani, Aila Sarkesh, Pedram Ghaderi Shadbad, Ali Aghebati-Maleki, Leili Aghebati-Maleki

**Affiliations:** 1 Student Research Committee, Tabriz University of Medical Sciences, Tabriz, Iran; 2 Department of Veterinary, Medicine, Tabriz Branch, Islamic Azad University, Tabriz, Iran; 3 Stem Cell Research Center, Tabriz University of Medical Science, Tabriz, Iran; 4 Immunology Research Center, Tabriz University of Medical Sciences, Tabriz, Iran; 5 Department of Immunology, School of Medicine, Tabriz University of Medical Sciences, Tabriz, Iran

**Keywords:** Multiple sclerosis, Experimental autoimmune encephalitis, EAE, Immune checkpoint, Immunotherapy

## Abstract

Beyond the encouraging results and broad clinical applicability of immune checkpoint (ICP) inhibitors in cancer therapy, ICP-based immunotherapies in the context of autoimmune disease, particularly multiple sclerosis (MS), have garnered considerable attention and hold great potential for developing effective therapeutic strategies. Given the well-established immunoregulatory role of ICPs in maintaining a balance between stimulatory and inhibitory signaling pathways to promote immune tolerance to self-antigens, a dysregulated expression pattern of ICPs has been observed in a significant proportion of patients with MS and its animal model called experimental autoimmune encephalomyelitis (EAE), which is associated with autoreactivity towards myelin and neurodegeneration. Consequently, there is a rationale for developing immunotherapeutic strategies to induce inhibitory ICPs while suppressing stimulatory ICPs, including engineering immune cells to overexpress ligands for inhibitory ICP receptors, such as program death-1 (PD-1), or designing fusion proteins, namely abatacept, to bind and inhibit the co-stimulatory pathways involved in overactivated T-cell mediated autoimmunity, and other strategies that will be discussed in-depth in the current review.

Video Abstract

**Supplementary Information:**

The online version contains supplementary material available at 10.1186/s12964-023-01289-9.

## Introduction

Multiple Sclerosis (MS) is a heterogenous chronic neurodegenerative disease defined by autoreactive immune invasion, peripherally mediated inflammation, and persistent central nervous system (CNS)-compartmentalized inflammation, which leads to demyelination and increasing disability [1]. Case reports from the 15th and 16th centuries, usually in the form of diaries, attest to the disease's long history. In 1868, neurologist Jean-Martin Charcot was the first to precisely characterize the features of MS, with Charcot's Triad referring to three significant MS symptoms, including nystagmus, intention tremor, and scanning speech. Extensive research and modern epidemiological data collection have been carried out since the 1940s [2]. MS affects approximately 2.8 million people worldwide, with women expected to have it twice as often as men on a global scale; however, the gender ratio in certain countries reaches 4:1 [3]. Even in formerly low-prevalence areas, MS incidence and prevalence are still on the rise, and it has been demonstrated that this trend is positively correlated with the growing female-to-male ratio [4]. MS’s clinical manifestations and course vary; in most cases, recurrent episodes of neurological impairments that often recover within days or weeks define the disease's early stages, known as relapsing-remitting MS (RRMS). Over time, it can be followed by secondary progressive MS (SPMS), which is characterized by the development of permanent neurological impairments and disability progression induced by neurodegeneration-associated microglia activation and disease deterioration. Then, there's primary progressive MS (PPMS), which begins and progresses gradually but is more hazardous, accounting for a lower proportion of MS patients [5, 6].

Breakthroughs in the management of vascular comorbidities, as well as a robust armamentarium of disease-modifying treatments (DMTs), have increased the chances of MS patients reaching stable disease and survival [7, 8]. Although immunotherapies that target the disease's inflammation have contributed to dramatic mitigation in CNS lesion formation and relapse rate, these treatments are largely insufficient to avert the formation of permanent disability caused by axonal and neuronal damage and loss, especially during the disease's progressive phase, suggesting an imperative need for further research into efficient immunomodulatory therapies [9].

The development of demyelinating plaques in the white and grey matters of the brain and spinal cord is the pathological hallmark of MS. MS etiopathogenesis is predominantly driven by dysregulated Th1 and Th17 mediated autoreactive responses elicited by environmental pathogens or other stimuli harboring antigenic sequences similar to those found in myelin in genetically susceptible individuals, culminating in molecular mimicry and cross-reactivity with myelin, followed by immune cell infiltration and localized inflammatory CNS damage [10]. As major regulators of immune tolerance, immune checkpoints (ICPs) play a fundamental role in maintaining the amplitude and duration of T-cell responses by regulating the equilibrium of costimulatory and co-inhibitory signaling pathways.

Given the above, strategies to target ICPs, whether to induce inhibitory ICPs or block stimulatory ICPs, have gained interest in investigations of various autoimmune diseases, notably MS or its animal model known as experimental autoimmune encephalomyelitis (EAE), with promising outcomes. The purpose of this review is to discuss the findings on the pathological implications of both inhibitory and stimulatory ICPs, as well as the efficacy and perspectives of ICP-based immunotherapy in the treatment of MS.

## Inhibitory immune checkpoints in MS

### PD-1

The inhibitory ICP receptor programmed cell death 1 (PD-1) is expressed on activated T cells in infections, malignancies, and autoimmune diseases. Its ligands include PD-L1, which is found on both hematopoietic and non-hematopoietic cells, and PD-L2, which is exclusively expressed on antigen-presenting cells (APCs) and can be induced by inflammatory signals [11, 12]. Lymphatic vessels in the meninges have been discovered to provide a drainage pathway for PD-L1^+^ immune cells from the cervical nodes to the brain [13].

Single-nucleotide polymorphisms (SNPs) have been under the spotlight by scientists for several years now. Some studies have indicated an association between SNPs in *PDCD1* and MS [14, 15]. In this respect, PD-1 expression on T cells from patients with different types of MS was analyzed, and it was revealed that a polymorphism of an intronic +7146G/A of PD-1 didn't affect MS susceptibility, but there was a notable aggravation of ongoing MS. After assessing the function of the protein encoded by the mutated gene, it was demonstrated that T cells expressing this polymorphism of PD-1 secreted a lower amount of IFN-γ. As well, mutations in the Runt-related transcription factor 1 (RUNX-1) binding site in the PD-1 gene resulted in defective PD-1-mediated inhibition of IFN-c production in MS patients [14]. In contrast, a case-control study of Iranian MS patients and healthy controls evaluating the frequency of different genotypes and alleles of PD-1.3 (+7146 G/A) demonstrated that the control group had a higher frequency of allele A and genotype AA [16]. Nonetheless, another study on an Iranian population found no significant link between PD-1.3 SNPs and MS risk [17]. Another case-control study on a Polish population suggested that PD-1 polymorphisms seem to be more disease-modifying than a risk factor for MS. They found the exclusive presence of a specific PD-1 haplotype containing the PD-1.3 A variant (PD-1.3A/PD-1.5T/PD-1.9C) in patients with a non-severe outcome. Furthermore, PD-1.5T alleles were associated with early signs of the disease, promoting pyramidal signs while protecting against diplopia. Similarly, diplopia development was observed to be more prevalent in patients harboring the PD-1.3G/PD-1.5C/PD-1.9C haplotype, but it was the reverse in patients harboring the GTC haplotype [15].

Analysis of peripheral blood mononuclear cells (PBMCs) from MS patients revealed a downregulated expression profile of PD-1 and PD-L1 mRNA that was substantially correlated with disease progression and the expanded disability status scale (EDSS) score. However, no remarkable difference was detected in the PD-L2 mRNA levels between MS patients and healthy controls [18, 19]. Likewise, MS patients were shown to have dramatically higher PD-1 expression in CD4^+^CD25^bright^ FoxP3-exon^2+^ Treg cells compared to healthy controls, contributing to the dysfunction and exhaustion of these cells [20]. Furthermore, *ex vivo* functional assessments of Th17 and Th1 clones from MS patients revealed that Th17 cells are less susceptible to suppression and have a higher proliferative capacity, which could be attributed to increased expression of co-stimulatory molecules, namely PD-1, in Th17 cells compared to Th1 cells [20]. It was also discovered that the proportion of T follicular helper (Tfh) cells, PD-1^+^ Tfh cells, and PD-1^+^ inducible costimulator (ICOS)^+^ Tfh cells increased in the cerebrospinal fluid (CSF) of MS patients, with PD1^+^ Tfh cells showing a positive correlation with the number of CSF plasma cells and disease progression [21]. Additionally, research has demonstrated that the frequency of CD3^+^ CD8^+^ T cells in MS patients is significantly lower than in healthy controls; however, these cells were shown to overexpress PD-1 in correlation with Epstein-Barr virus (EBV) load, suggesting a pathogenic implication of EBV in MS development and exacerbation [22]. Consistent with these findings, studies on CD8^+^ CD57^+^ T cells in MS patients, which act as effector cytotoxic cells against EBV-infected cells, revealed that PD-1 expression impairs degranulation and cytotoxicity in these cells, compromising the immune control of EBV during the remission phase and contributing to disease exacerbation [23]. Conversely, the decreased frequency of PD-1^+^ cells might indicate a distinct distribution and/or deficient generation of memory T cells, as has been postulated to underpin the defective control of EBV by CD8^+^ T cells in MS [24]. CD8^+^ PD-1^+^ T cells constitute a significant proportion of T cells found in MS lesions, as well as clusters of CSF CD8^+^ T cells correlated with MS, with the latter being more frequent in the CNS than in peripheral blood [25–28]. Although inflammatory conditions induce PD-L1 expression in microglia, astrocytes, and infiltrating mononuclear cells [29], pathological studies on post-mortem brain tissues confirmed that more than half of infiltrating CD8^+^ T cells lack PD-1 expression and are thus insensitive to PD-L1/L2, pointing to a potential therapeutic implication of PD-1 augmentation for MS patients [30]. On the other hand, analysis of human brain endothelial cells found that these cells are positive for PD-L2 in both MS and healthy individuals, with MS patients having roughly half the expression of healthy controls, which contributes to defective regulation of T cell transmigration to the brain [31]. Further, several researchers have suggested that the expression pattern of PD-1/PD-L1 differs depending on the disease state. In this way, the frequency of PD-1^+^ CD4^+^ T cells, PD-1^+^ CD8^+^ T cells, PD-L1^+^ interleukin (IL)-10^+^ CD14^+^ cells, and PD-L1^+^ IL-10^+^ CD19^+^ cells in MS patients were found to be notably higher in the remitting phase compared to the relapsing phase [32]. Also, the frequency of circulating Tfh cells (CD3^+^CD4^+^CXCR5^+^PD-1^+^) in relapsing RRMS patients was shown to be increased in comparison to remitting RRMS and healthy controls [33]. PD-L1 overexpression in APCs was also demonstrated to reduce autoreactive T cell immunity in EAE mice, and PD-L1 deficient EAE models displayed significantly worse symptoms. These were accompanied by significant increases in tumor necrosis factor (TNF)-α, IL-17, and IFN-α production [34–36], all of which trigger severe inflammatory responses that are critical in the pathophysiology of MS and EAE [37]. Additionally, it has been outlined that during the acute phase of EAE, elevated expression of PD-L1 in the CNS results from overexpression on resident microglia and infiltrating myeloid PD-L1^+^ major histocompatibility complex (MHC) class II^high^ APC, contributing to suppression of IFN-γ responses of activated Th1 cells rather than Th17 cells, whereas PD-L2 is mostly present on a subset of infiltrating myeloid DC and macrophages [38]. On the other hand, PD-L1 expression is higher in MS plaques than in non-pathological CNS tissues. It was consistently shown to be abundantly expressed in inflammatory regions of white matter in MS patients, and it was believed to be expressed on activated microglia/macrophages [39].

Several studies have investigated the microRNA (miRNA/miR)-mediated regulation of PD-1/PD-L1 in MS and EAE. miRNAs are endogenous non-coding RNAs that are involved in the post-translational regulation of gene expression by inhibiting or degrading their corresponding messenger RNA (mRNA), the aberrant expression profile of which has been evidenced in plethora of autoimmune diseases and malignancies [40–42]. Among the dysregulated miRNAs found in MS and EAE, upregulation of miR-16, miR-142-3p, and miR-155 have been shown to facilitate the downregulation of *PDCD1* as a target gene [43, 44], and blocking both miRNAs and *PDCD1* can affect each other's expression. In this respect, miR-155 depletion in mice was found to be protective against EAE, even though, PD-1 blockade restored its susceptibility to EAE [45]. Similarly, diminished expression of miR-1, miR-20a, miR-28, miR-95, miR-146a, miR-335, and miR-625 in PBMCs of pregnant MS patients was discovered to upregulate their targets PD-L1 and PD-L2, potentially contributing to a lower recurrence rate of MS in the pregnant population [46].

Exacerbation of disease and increased neurological severity have been documented in EAE mice following PD-1 blockade [34]. PD-1 interaction with PD-L1, unlike PD-L2, can protect against EAE, as demonstrated by the observation that PD-1^-/-^ and PD-L1^-/-^ mice had more severe EAE than wild type and PD-L2^-/-^ mice, and PD-1^-/-^ and PD-L1^-/-^ cells exhibited higher production of the proinflammatory cytokines IFN-γ, TNF, IL-6, and IL-17 [36]. PD-1 blockade in EAE was also shown to activate antigen-specific T cells and their inflammatory cytokine production [34]. Similarly, PD-1 depletion in lymphocytes derived from MS patients during the acute phase was associated with a proliferative effect on CD4^+^ and CD8^+^ T cells; nevertheless, these consequences were not evidenced while PD-L1 on the APCs was suppressed, suggesting that PD-1 has a more prominent role than PD-L1 in inducing lymphocyte apoptosis and enhancing lymphocyte proliferation in MS [47].

PD-1/PD-L1 was also proposed to underpin the mechanism of estrogen (E2) protection against EAE since the lack of PD-1 on B cells in E2-implanted mice was found to enhance activated CD4^+^ T cell frequency while decreasing the frequency of Bregs [48]. Nevertheless, it exhibits a protective role against EAE through PD-L1 upregulation on B cells and regulatory T cells [49, 50]. Despite this, PD-L1 suppression in another study had no significant effect on the onset and severity of EAE [51]. Additionally, E2 has been shown to elevate intracellular PD-1 expression in FoxP3^+^ Tregs while significantly decreasing IL-17 production in the periphery of EAE-induced mice [52]. Although PD-1 knockout was coupled with normal Foxp3 expression in Tregs, suppressive activity was markedly impaired, showing that PD-1 expression plays a vital role in E2-mediated Treg immunosuppression [53]. E2 also plays a key role in immunosuppression in EAE mice through the PD-L1 upregulation of monocytes and macrophages in the periphery and CNS [54]. As well, it increases PD-L1 expression on DCs, which additionally enhances the formation of tolerant DCs and dampens the development of EAE [55]. Furthermore, several currently used DMTs, including Fingolimod [56], Siponimod [57], and IFN-β [28] were demonstrated to induce PD-1 expression on Tfh, Treg, and CD8^+^ T cells, respectively.

Given the pathogenic implications of PD-1/PD-L1 suppression in the development of MS and EAE, multiple investigations have been conducted to boost this pathway as a therapeutic strategy (Table 1). In this respect, Hirata et al. transfected embryonic stem cell-derived DCs with PD-L1 and administered them intraperitoneally, enabling them to establish immune tolerance and reduce the severity of myelin oligodendrocyte glycoprotein (MOG)-induced EAE [58]. Consistently, Chang et al. utilized 5-aza-2′-deoxycytidine (5-aza) to induce promoter hypomethylation of PD-L1 in DCs, which culminated in suppressed CD4^+^ T cell activation and thereby inhibited EAE progression [59]. Similar to this, estriol (E3) treatment of DCs (E3 DCs) could upregulate the expression of inhibitory costimulatory markers, particularly, PD-L1/L2, creating tolerogenic DCs. E3 DCs were then administered to EAE mice before the disease was actively induced, and this exerted protective effects by reducing the production of proinflammatory cytokines IL-12, IL-23, and IL-6 and increasing anti-inflammatory cytokines IL-10 and transforming growth factor beta (TGF-β) [55]. Additionally, it was discovered that 1,25(OH)_2_D_3_ treatment of DCs to create tolerogenic DCs (VD3-DCs) upregulated PD-L1 on these cells; thus, the transfer of these cells enhanced PD-1 expression of spleen and lymph node T cells and alleviated the clinical symptoms of EAE [60]. Nonetheless, another study found that intracerebral administration of PD-L1-knocked-out DCs was coupled with the recruitment of regulatory CD8^+^ T cells to the CNS, resulting in EAE amelioration [61]. Likewise, the adoptive transfer of a novel subtype of Treg cells with high expression of PD-L1 was shown to suppress the EAE in close correlation with PD-L1 expression [62]. Similar findings were observed when Breg cells with abundant expression of PD-L1 were adoptively delivered to the EAE mouse model, which led to the diminished release of the inflammatory cytokines IFN-γ and IL-17 [63]. Moreover, IL-12 treatment stimulates the production of the Th1 cytokine IFN-γ, which drives upregulation of PD-L1/L2 expression at both mRNA and protein levels on CD11b^+^ APCs and, ultimately, contributes to the inhibition of EAE [64]. Besides that, MIS416, a novel immunomodulatory activator of nucleotide-binding oligomerization domain-containing protein 2 (NOD-2) and Toll-like receptor 9 (TLR9) signaling, was shown to reduce immune infiltration into the CNS and alleviate EAE severity via a variety of mechanisms, including raising the frequency of red pulp macrophages and inducing IFN-γ-mediated PD-L1 upregulation on these cells, while promoting the homeostatic tracking of PDL-1-expressing myeloid subsets into the CNS [65]. Augmentation of PD-1, PD-L1, and PD-L2 expression in the spinal cord was also demonstrated in EAE mice treated with Astragalus polysaccharides (APS), an active extract derived from Astragalus membranous, which was coupled with EAE remission [66]. Further, Herold et al. suggested that early delivery of PD-L1 Ig fusion protein for five consecutive days was linked to a long-lasting amelioration in the severity of the disease by inhibiting the development of Th17 cells and the related transcription factor retinoic acid-related orphan receptor (ROR)-γt as well as interferon-regulatory factor 4 (IRF4) [67].

**Table 1:** Immune checkpoint-based immunotherapies in EAE models

**Target ICP**	**Intervention**	**Outcome**	**Reference**
**PD-1**	PD-L1^high^ DC	Gene-modified DC: It reduced T-cell response to MOG, cell infiltration into the spinal cord, and EAE severity. 5-aza-treated DC: It inhibited CD4^+^ T cell proliferation and cytokine secretion, inflammatory cell infiltration, CNS demyelination, and EAE progression. E3-treated DC: It protected against developing EAE through immune deviation to a Th2 response. 1,25(OH)_2_D_3_-treated DC: It enhanced PD-1 expression of spleen and lymph node T cells and alleviated the clinical symptoms of EAE.	[55, 58–60]
	PD-L1-knocked out DC	The treatment enhanced the recruitment of regulatory CD8^+^ T cells to the CNS and EAE amelioration.	[61]
	PD-L1 ^high^ Treg cell	The treatment inhibited EAE in a FoxA1-and PD-L1-dependent manner.	[62]
	PD-L1 ^high^ Breg cell	The treatment protected against the development and severity of MOG-induced EAE and diminished the release of inflammatory cytokines IFN-γ and IL-17.	[63]
	IL-12	The treatment inhibited EAE by upregulating PD-L1 on CD11b^+^ APCs via an IFN-γ-dependent manner.	[64]
	MIS416	The treatment prevented EAE via IFN-γ-dependent expansion of PD-L1-expressing peripheral myeloid cells.	[65]
	PD-L1 Ig fusion protein	The treatment led to long-lasting amelioration in the severity of EAE by inhibiting the development of Th17 cells and the ROR-γt as well as IRF4.	[67]
**CTLA-4**	CTLA-4-Fc	The treatment resulted in significant recovery after an acute episode, EAE relapses, and full clinical remission while having no notable impact on the rate of EAE relapse. The treatment also protected against EAE through anti-inflammatory effects and prevented demyelination or axonal loss.	[68, 69]
	dNP2-ctCTLA-4	The treatment led to decreased Th1 and Th17 cell infiltration to the CNS and demyelination. The treatment also led to EAE remission with long-term control and prevention of relapse through expansion of Foxp3+ Tregs, Foxp3 expression during Th1 or Th17 cell differentiation, and CTLA-4 expression.	[70, 71]
	B7-1 Ig	The treatment ameliorated EAE through the development of naïve MBP-specific Th precursor cells via the Th2 pathway.	[72]
**CD40**	Anti-CD40L Ab	The treatment at either the peak of acute EAE or during remission inhibited clinical disease progression and CNS inflammation by suppressing Th1 differentiation and effector function, IFN-γ release, myelin peptide-specific delayed-type hypersensitivity responses, as well as inducing encephalitogenic effector cells. The treatment also mitigated EAE severity by regulating IL-10- and IL-35-producing Foxp3^+^ Treg cells.	[73, 74]
	KGYY_6_	The treatment prevented the progression of the disease and alleviated symptoms through binding to Th40 and memory T cells and upregulating the expression of CD69 and IL-10 in the CD4^+^ T cell compartment.	[75]
**CD137**	Agonistic anti-CD137 Ab	The treatment reduced EAE incidence and severity through suppression of IFN-γ-releasing CD8^+^ T cells, Th17 cells, and related pathogenic IL-17 release while raising Foxp3^+^ CD4^+^ Treg cell frequency in an IFN-γ-independent manner. Even though, this was only effective when administered during the disease induction phase.	[76, 77]
	CD137L knockout	The treatment protected against demyelination and the development of EAE by restricting encephalitogenic T cell activation and inflammatory cytokine release, as well as their trafficking into the CNS by downregulating VCAM-1.	[78]

*Abbreviations*: *Ab* Antibody, *Breg* Regulatory B cell, *CNS* Central nervous system, *CTLA-4* Cytotoxic T-lymphocyte-associated protein 4, *DCs* Dendritic cells, *dNP2-ctCTLA-4* Cell-penetrating peptide-conjugated cytoplasmic domain of CTLA-4, *EAE* Experimental autoimmune encephalomyelitis, *Foxp3* Forkhead box P3, *ICP* Immune checkpoint, *IFN* Interferon, *IL* Interleukin, *IRF4* Interferon regulatory factor 4, *MBP* Myelin basic protein, *MOG* Myelin oligodendrocyte glycoprotein, *PD-1* Programmed cell death protein 1, *PD-L1* Programmed death-ligand 1, *ROR-γt* Retinoic acid-related orphan receptor gamma t, *Th* T helper, *Treg* Regulatory T cell, *VCAM-1* Vascular cell adhesion molecule 1

Overall, the PD-1/PD-L1 pathway shows promise as a therapeutic target for MS, but further research is necessary to fully comprehend its pathological implications and develop safe and effective clinical approaches. This entails determining the optimal administration method and dosage for PD-1/PD-L1 modulators, evaluating potential side effects, and understanding how the pathway interacts with other immunomodulatory pathways. Conducting clinical trials is essential to establishing the safety and efficacy of PD-1/PD-L1 pathway modulators in MS patients. Despite the challenges involved, leveraging PD-1/PD-L1 pathway modulation as an MS treatment holds significant potential benefits and could result in a more precise and efficient therapeutic approach with fewer adverse effects compared to current treatments. Ongoing research in this field is critical for the development of safe and effective PD-1/PD-L1 pathway modulators for MS treatment.

#### VISTA

V domain-containing Ig suppressor of T-cell activation (VISTA), a member of the B7 family, is a one-of-a-kind inhibitory ICP broadly expressed on CD4^+^ T cells, CD8^+^ T cells, neutrophils, DCs, and monocytes [79]. It has two ligands: P-selectin glycoprotein ligand 1 (PSGL-1) and V-set and Ig domain-containing 3 (VSIG3), the latter of which is expressed exclusively on neurons and glial cells in the brain and on Sertoli cells in the testis [80], and its interaction with VISTA inhibits the release of IFN-γ, IL-2, and IL-17 cytokines [81]. VISTA, as a co-inhibitory ligand expressed on APCs, can suppress T cells while also acting as a co-inhibitory receptor on CD4^+^ T cells, suggesting that an aberrant expression profile of VISTA results in the induction of over-activated T cell immunity [82]. In VISTA-deficient mouse models, the increase of downstream chemokines induced by IFN-γ, such as interferon-gamma-induced protein 10 (IP-10) and monocyte chemoattractant protein-1 (MCP-1) supports the inhibitory implications of VISTA in Th-1 polarization [83]. Also, the analysis of T cell compartments in VISTA knockout mice using transcriptome and epigenetic techniques indicated that VISTA is a critical factor in the maintenance of naïve T cell quiescence. T cell tolerance is therefore regulated before activation, whereas other ICPs such as CTLA-4 and PD-1 act after T cell activation. Thus, VISTA is considered a pioneer and a novel therapeutic target since it is the first known ICP to act in the initial stages of T cell activation [84]. In this way, VISTA suppression in aged mice led to spontaneous stimulation of CD8^+^ and CD4^+^ T cells and loss of tolerance [83], implying the significance of VISTA in autoimmunity, as its knockdown in autoimmune diseases, including systemic lupus erythematosus, was found to exacerbate the disease [85]. Similarly, inflammation and tissue damage in EAE deteriorate as a consequence of infiltrating T cells' increased activation triggered by VISTA downregulation on microglia cells in lesions [86].

Studies on EAE models have shown that VISTA knockout mice are more susceptible to EAE by increasing the amount of IFN-γ and IL-17A-producing CD4^+^ T cells in the CNS [83, 87]. In line with these findings, Borggrewe et al. found that VISTA is expressed differentially in microglia during inflammation and neurodegeneration, establishing the first evidence of VISTA activity in microglia during CNS pathology. They isolated microglia from EAE mice to investigate the changes in microglial VISTA expression during CNS inflammation. In comparison to nonimmunized mice, VISTA expression was significantly lower in all stages of EAE, including scores 1, 4, and remission, in the forebrain, spinal cord, and hindbrain microglia. Furthermore, VISTA expression was shown to be dramatically reduced in chronic MS lesions due to high activation of microglial cells, while it was abundantly expressed in microglia cells in the normal-appearing white matter, where they were not active [88]. Chronic MS lesions were shown to have dysregulated macrophage and lymphocyte activity, as well as extensive immune cell infiltration [89], which may facilitate the downregulation of VISTA expression, leading to greater infiltration [90]. So, according to the association between VISTA expression pattern and immune infiltration in the periphery of MS, it's reasonable to infer that VISTA expression in activated microglia in CNS pathologies may be influenced by external variables. Besides, decreased VISTA levels in microglia may hamper phagocytosis, which is a critical mechanism for eliminating cellular debris early in the disease [91]. Further functional evaluations of microglial VISTA in MS lesions revealed that VISTA has a differential expression in various stages of MS lesions, with the lowest expression during inflammation, and its knockout can stimulate morphological and transcriptional alterations toward an immune-activated and proliferative profile with amoeboid morphology and overexpression of genes involved in TNF and IFN signaling as well as the cell cycle. Besides, microglial VISTA was found to be implicated in the induction of a regulatory and homeostatic microglia phenotype that intactly uptakes myelin [92], which would otherwise contribute to oligodendrocyte damage through antigen presentation to infiltrating T cells [93].

Borggrewe et al. also observed the downregulation of VISTA expression in endothelial cells in chronic MS lesions. Endothelium involvement in MS pathology comprises the chemotaxis of immune cells to the CNS [94] and their antigen presentation of CNS constituents to antigen-specific lymphocytes [95–97]. Lymphocyte activation and transmigration into the CNS can consequently be promoted due to reduced expression of VISTA on the endothelium [88]. As well, according to Derakhshani et al., investigations on PBMCs of RRMS patients demonstrated a significant downregulation of VISTA mRNA expression, and further single-cell RNA sequencing analysis of various cell populations confirmed a decreasing trend in the expression pattern of VISTA in monocytes, DCs, and naïve B-cells of untreated RRMS patients compared to healthy controls [98].

VISTA is one of the most recently found immunotherapy targets, and while our understanding of its biology in the CNS is limited, it is conceivable to develop novel therapeutic approaches for CNS diseases such as MS by either increasing or reducing its activity [86]. Immunoenhancing anti-VISTA antibodies (antagonists) and immunosuppressive anti-VISTA antibodies (agonists) are the two main strategies to silence or boost VISTA signaling, respectively, with the administration of the latter accompanied by decreased severity of inflammation in EAE, while VISTA antibodies exacerbated the disease [86, 87, 99]. Hence, agonistic antibodies targeting VISTA may be an effective therapy for MS [86]. Nonetheless, research on the therapeutic effects of currently used drugs for MS treatment, including IFNβ-1α, glatiramer acetate (GA), and dimethyl fumarate (DMF) has been shown to interfere with the VISTA expression, as the VISTA mRNA was found to be upregulated in PBMCs of the treated patients compared to their treatment naïve counterparts [98].

Collectively, the expression of VISTA has been consistently observed to be downregulated in MS and EAE, exhibiting distinct patterns across different stages of the disease. Despite the limited research investigating the precise involvement of VISTA in the pathogenesis of MS and EAE, emerging evidence indicates a promising potential for augmenting VISTA expression as a viable therapeutic target. Nevertheless, further rigorous examination through preclinical and clinical studies is imperative to validate these findings, thereby establishing the conclusive role of enhancing VISTA expression as an efficacious therapeutic strategy for MS patients.

### LAG-3

Lymphocyte-activation gene 3 (LAG-3) is an essential ICP that has been implicated in cancer, infectious disease, and autoimmunity. However, much remains unknown about its mechanism of action. LAG-3 is a member of the immunoglobulin superfamily and the product of a gene duplication event [100]. LAG-3 presumably inhibits the activation of the host cell and enhances immune response suppression [101]. It is mostly investigated in Tregs and conventional T-cells, though it is also expressed in natural killer (NK) cells, neurons, B-cells, and plasmacytoid dendritic cells (pDCs) [102–105].

Clinicopathological studies have shown a significant correlation between LAG-3 expression, MS susceptibility, and the disease course of MS. It has been shown that SNPs in LAG-3 confer a moderate risk for MS development [106]. Likewise, rs1922452 of the LAG-3 gene in Jordanian MS patients was found to be correlated with MS comorbidity [107]. However, another SNP genotyping study on two large sample sets of Swedish MS patients and healthy controls failed to establish any association between the LAG-3 gene and MS [108]. Besides, LAG-3 mRNA expression in PBMCs derived from MS patients was found to be significantly greater than their control counterparts at baseline and during the disease course, and it serves as an adverse prognostic factor [109]. Similarly, Jones et al. discovered a global dysregulation of LAG-3 expression on CD4^+^ and CD8^+^ T cells from RRMS patients due to reduced LAG-3 transcription, which induced enhanced T cell proliferation and persistence as well as lower expression of apoptosis markers and resistance to cell death. Their findings emphasized the significance of a LAG-3 agonist in the treatment of autoimmunity [110]. On the other hand, studies on EAE models have shown that LAG-3 deficiency in CD4^+^ FoxP3^+^ Tregs in the CNS enhances IFN-γ and granzyme B production, which, in contrast to Tregs' protective role in maintaining immune tolerance, shifts them toward a contributor to MS pathogenesis [111].

Although the precise role of LAG-3 in EAE and MS pathology remains elusive, available evidence indicates its contribution to disease susceptibility and progression. Further investigation is necessary to fully understand the underlying mechanisms involved and to explore the potential of LAG-3 as a therapeutic target for MS.

#### TIM-3

The receptor for T-cell immunoglobulin and mucin-containing protein-3 (TIM-3) is expressed on DCs, macrophages, T cells, and NK cells. It contributes to the development and maintenance of immune tolerance by inducing T-cell apoptosis or by innate immune cell suppression [112, 113]. At least four ligands have been discovered for this ICP: carcinoembryonic antigen-related cell adhesion molecule 1 (Ceacam-1) [114], high mobility group protein B1 (HMGB1) [115], Galectin-9 (Gal-9), and Phosphatidyl serine [116]. It’s proven that blocking TIM-3 on NK cells enhances IFN-γ production [117].

Several studies have shown a correlation between TIM-3 SNPs and MS susceptibility [118–120]. For example, a case-control study of the Iranian population discovered a significant link between TIM-3 SNPs and MS susceptibility, with -574 and -1516 C>A SNPs in the promoter region of the TIM-3 gene posing a risk for MS development, while A/C genotypes for the -574 and -1516 loci of the TIM-3 gene were significantly lower in MS patients [118]. Another study on CD4^+^ T cell clones derived from MS patients' CSF revealed that these cells released more IFN-γ than control subjects, exhibited downregulated expression of T-bet and TIM-3, and when polarized under Th1 conditions that evoke T-bet and TIM-3 expression, clones from control subjects upregulated TIM-3 at much higher rates than T cell clones from MS patients [121]. Similarly, investigations on CD4^+^ T cells from MS patients and control subjects revealed a significant functional implication for TIM-3 expression in CD4^+^ T cell IFN-γ production; however, unlike in control subjects, monoclonal antibody (mAb)-mediated TIM-3 blockade did not result in increased IFN-γ production in CD4^+^ T cells from MS patients. Interestingly, when MS patients were given GA or IFN-β, the defective immunoregulation of TIM-3 was reversed, and the increase in IFN-γ production of CD4^+^ T cells in both groups of control and MS patients was comparable, suggesting that TIM-3 regulation is a therapeutic mechanism of these drugs [122]. GA has also been discovered to bind the integrin macrophage-1 antigen (CD11b/CD18) and upregulate TIM-3 mRNA in PBMCs, leading to promoted phagocytosis of monocytes in MS patients [123]. Furthermore, TIM-3- and Gal-9-blocking antibodies were shown to mitigate apoptotic cell death of myelin basic protein (MBP)-specific T lymphocytes while boosting IFN-γ and IL-17 expression in benign MS, RRMS, and healthy control groups, though not in the PPMS group, demonstrating the importance of TIM-3 expression pattern in determining the clinical phenotype of the disease [124]. The same scientists obtained similar results by examining the relationship between the CEACAM-1/TIM-3 pathway and the clinical type of MS patients [125]. However, in contrast to these findings, an *ex vivo* analysis of Th1 and Th2 cells, as well as mononuclear cells from the CSF of MS patients, revealed a differential expression pattern of TIM molecules. Specifically, Th1 cells overexpressed TIM-3, which is associated with higher IFN-γ and TNF-α expression, while Th2 cells overexpressed TIM-1, which is associated with lower IFN-γ and TNF-α expression. This differential expression pattern was found to be associated with the clinical state of the disease [126]. Despite these discrepancies, multiple studies support TIM-3's undeniable role in Th1 response down-regulation, suggesting its significance in suppressing effector Th1 cells during normal immune responses and inducing peripheral tolerance [127–129].

In summary, the current understanding of the pathologic implications of TIM-3 in MS pathogenesis is still emerging. Some studies have consistently demonstrated the downregulation of TIM-3 in MS patients, indicating its potential involvement in the disease. Therefore, developing therapeutic strategies to enhance TIM-3 expression may represent a promising approach for MS treatment. Further preclinical and clinical studies are required to determine the most effective way of targeting TIM-3 and to evaluate the safety and efficacy of such approaches.

#### CTLA-4

Cytotoxic T-lymphocyte antigen-4 (CTLA-4), a CD28 homolog, is an ICP receptor mostly expressed on DCs and activated T-cells including memory and regulatory T cells [130]. Its two ligands are CD80 (B7-1) and CD86 (B7-2). CTLA-4 is known to have an intracellular mechanism where downstream signaling occurs by binding to its ligands, however, the extracellular mechanism of action has been the subject of further investigation [131, 132]. According to immunological studies, CTLA-4 has an essential role in the induction of peripheral tolerance and inhibition of T cell function and proliferation when upregulated following T cell activation [133].

In the largest case series investigating clinical phenotypes of CTLA-4 deficiency, neurological complications, including autoimmune encephalomyelitis or encephalitis, were reported in 28% of subjects [134]. Likewise, increased expression of CTLA-4 was considered protective in experimental allergic encephalomyelitis [135].

Studies on the correlations between CTLA-4 SNPs and MS susceptibility have yielded conflicting results, with the majority of them failing to demonstrate any correlation [136–140]. For example, CTLA-4 A/G + 49 has been linked to MS susceptibility in several populations [141–143], while no correlation has been identified in others [144, 145]. Likewise, a study by Palacios et al. discovered a decline in CTLA-4 isoforms expression associated with some alleles of SNP 658 in MS patients, but they inferred that the epigenetic alterations are mainly regulated by the disease itself and the implication of CTLA-4 in MS is roughly attributable to functional changes in its pathway [146]. Some researchers also analyzed the significance of CTLA-4 SNPs as a prognostic factor for MS progression and the clinical course of the disease. In this way, Karabon et al. demonstrated that CTLA-4 gene polymorphisms CT60A and Jo31T, which have a positive correlation with membrane and cytoplasmic CTLA-4 expression of CD4^+^ T cells, enhanced the risk of paresthesia and pyramidal signs as the first manifestation of disease, as well as an earlier transition to SPMS in RRMS patients who were carriers of these alleles [147]. As well, Mäurer et al. found that PPMS patients were more likely to be carriers of the +49 G allele than patients with about onset of disease [148]; however, this increasing trend of carriage for this allele in PPMS patients compared to RRMS patients was not shown to be significant in another study by Heggarty et al [143]. Furthermore, pathological analysis of MS lesions has identified an rs5742909/CTLA-4 polymorphism that is associated with diminished remyelination [149]. Consistently, next-generation sequencing (NGS) analysis of a case of MS revealed that the patient was heterozygous for a potentially pathogenic frameshift deletion variation in CLTA-4 exon 1:c.81dup p.(leu28Serfs*32), which was associated with CNS inflammatory demyelination [150].

Several *ex vivo* analyses of CTLA-4 expression on CD4^+^ T cells, CD8^+^ T cells, monocytes, and PBMCs derived from treatment-naive MS patients and healthy counterparts did not reveal a significant difference between the two groups [109, 151, 152]. According to a study by Mena et al., patients with a rapidly progressing disease were the only group with a significantly increased expression profile of CTLA-4 on CD4^+^ and CD8^+^ T cells [151]. Conversely, other studies found CTLA-4 downregulation in RRMS patients compared to healthy controls [19, 153]. On the other hand, it was shown that the proportion of CTLA-4^+^ CD4^+^ T cells in treatment-naive RRMS and SPMS patients was substantially higher than that in healthy controls and that this difference was more pronounced in RRMS patients. Additionally, when CD4^+^ T cells from study subjects were stimulated with anti-CD3^+^ rIL-2, CD4^+^ T cells from SPMS patients were unable to induce normal surface CTLA-4 expression, and even among RRMS patients, its expression was decreased [154]. Similarly, CTLA-4 blockade in MBP-reactive T cells from MS patients and healthy controls during stimulation resulted in a proliferative and enhanced cytokine response, whereas this effect was much lower in MS patients, inferring that CTLA-4's regulatory function in MS patients might be compromised [152]. Also, a CSF analysis of PPMS and RRMS patients for the expression of CTLA-4 on memory CD8^+^ T cells found substantial age-related variations in CTLA-4 expression pattern, with a tendency for it to decline in healthy controls while being entirely abrogated in MS patients. In contrast to elderly patients, this aberration was notably evidenced in young MS patients, especially in patients with PPMS, implying a trend of premature immune aging in the CD8^+^ T cell compartment of young MS patients, with possible consequences for clinical outcomes [155].

In vivo, animal model investigations have shown controversies regarding the protective or detrimental consequences of CTLA-4 expression, with some studies demonstrating CTLA-4 deletion to protect against EAE induction [156–158] and others finding the reverse. In this way, Almolda et al. reported a differential CTLA-4 expression pattern during acute EAE, with CTLA-4^+^ cells being elevated in the recovery phase, regulating the termination of the immune response [159]. Mechanistically, activated microglia and macrophages in the CNS in the EAE mouse model have been demonstrated to be involved in 1,25(OH)_2_D_3_ synthesis and paracrine signaling to CNS-infiltrating CD4^+^ T cells, which upregulates CTLA-4 expression and protects against immune-mediated neurological damage [160]. Thus, vitamin D insufficiency, a well-known contributor to immune dysregulation in autoimmune and infectious diseases [161], may disturb the vitamin D3-regulated ICP in the prevention of MS. Similarly, several researchers investigated anti-CTLA-4 antibodies in EAE models and discovered that blocking CTLA-4 might aggravate inflammatory responses, autoreactivity, and clinical symptoms, as well as hinder the clinical remission of the disease [162–165]. Other researchers targeted CTLA-4 with a recombinant fusion protein constituted of the extracellular domain of human CTLA-4 bound to mouse IgG2a Fc (CTLA-4-Fc), which resulted in significant recovery after an acute episode, EAE relapses, and full clinical remission while having no notable impact on the rate of EAE relapse [68]. CTLA-4-Fc was also suggested to have protective and anti-inflammatory effects against EAE, with CTLA-4-Fc-treated animals showing essentially no demyelination or axonal loss as compared to their control counterparts [69]. Furthermore, conjugating the blood-brain barrier (BBB) permeable peptide dNP2 with the cytoplasmic domain of CTLA-4 (dNP2-ctCTLA-4) allowed for efficient delivery to EAE mice, resulting in decreased Th1 and Th17 cells infiltration to the CNS and demyelination [70]. Another study on dNP2-ctCTLA-4 in EAE models found that it can expand Foxp3^+^ Tregs, Foxp3 expression during Th1 or Th17 cell differentiation, and CTLA-4 expression, resulting in EAE remission with long-term control and prevention of relapse [71].

Interestingly, inhibiting CTLA-4 ligands displayed distinct consequences, as the interplay of B7-1 and B7-2 with their shared cognate receptors CD28 and CTLA-4 modulates the commitment of precursors to a Th1 or Th2 lineage, which influences the clinical outcomes. In this regard, anti-B7-1 Ig was shown to promote the development of naïve MBP-specific Th precursor cells via the Th2 pathway, whereas anti-B7-2 Ig was shown to contribute to Th1 cell development. Thus, the anti-B7-1 Ig treatment ameliorated EAE, while the anti-B7-2 Ig treatment resulted in clinical and histological deterioration of the disease [72].

Several investigations have also concentrated on the impact of current MS therapies on the CTLA-4 expression profile and function. In this way, Derakhshani et al. discovered that fingolimod, IFNβ-1α, and DMF could dramatically upregulate CTLA-4 expression in RRMS patients relative to treatment-naive patients, however, this effect was not observed in patients treated with GA [153]. Additionally, analysis of PBMCs from MS patients by Hallal-longo et al. demonstrated that patients treated with IFN-β had higher expression of intracellular CTLA-4, which culminated in diminished proliferative response to MBP and myelin and enhanced lymphocyte apoptosis [166]. Moreover, according to Sellebjerg et al., IFN-β treatment could elevate the frequency of CD25 ^high^ CD4^+^ T cells with CTLA-4 surface expression [167]. However, in vitro IFN-α or IFN-β treatment of PBMCs from RRMS did not result in changes in CTLA-4 mRNA expression [168]. According to a follow-up study by Espejo et al., there was no significant alteration of the T-lymphocyte proliferative response through the CD28/CTLA-4 pathway, within 3 months post-treatment in RRMS patients who received IFN-β. But after three months, it was shown that the CD80:CD28/CTLA-4 pathway was hindered by increased production of IL-10, resulting in impeded production of CD80-induced IL-2, which plays a key role in lymphocyte expansion and the development of autoimmunity [169].

Given the in vitro and in vivo evidence indicating the potential therapeutic implications of CTLA-4 targeting in MS, several clinical trials have been launched to evaluate them in the clinical setting (Table 2). In an open-label, phase 1 clinical trial, 16 RRMS patients received a single dose of intravascular CTLA Ig (RG2077, a recombinant CTLA4-IgG4m), followed by an extension study in which 4 additional patients received four doses of the therapy. The treatment was well tolerated, with just mild adverse effects, and the immunologic evaluation of the therapy after two months suggested a reduction in MBP proliferation and IFN-γ secretion by MBP-specific cells [170]. In another phase II placebo-controlled research, 65 RRMS patients were randomly allocated to receive abatacept (CTLA-4 Ig) or placebo for 24 weeks in a 2:1 ratio, then switched to the alternative therapy at 28 weeks and medicated with their final dose of the study treatment at 52 weeks. Although Abatacept was well tolerated, there were no substantial differences between the Abatacept and placebo groups in terms of the mean number of new gadolinium-enhancing (Gd+) MRI lesions, or other MRI and clinical parameters of the disease activity [171]. Nonetheless, Abatacept administration was found to drastically lower the relative frequencies of CD45RO^+^ Treg and Tfh cells in circulating CD4^+^ T cells as well as circulating plasmablasts as compared to placebo, while also suppressing their activity by downregulating activation markers CD38 and ICOS genes implicated in cell division and chromatin dynamics in these cells [172]. A case study was also conducted on a 14-year-old girl who presented with MS, immunodeficiency, enteropathy, splenomegaly, lymphadenopathy, and lymphocytic infiltration of non-lymphoid organs, as well as a confirmed heterozygous CTLA-4 mutation (c.208C>T, p.Arg70Trp). Abatacept was administered at a dose of 500 mg every four weeks (as indicated for weight range) and resulted in considerable improvements in MRI findings after two years, with no definite abnormal enhancing lesion in brain parenchyma observed in tandem with improvements in clinical symptoms. Flow cytometry analysis of the therapy response after two years showed greater CTLA-4 expression but no significant change in the frequency of Treg cells [173]. These findings corroborated the significance of integrating genetic analysis in the routine workup of MS patients to provide ICP-targeting medicines in a tailored manner.

**Table 2:** Clinical studies of immune checkpoint-based immunotherapies in MS patients

**Study design**	**Subjects**	**Target ICP**	**Intervention**	**Outcome**	**Reference**
Phase 1, open-label, clinical trial	16 RRMS	CTLA-4	RG2077 (recombinant CTLA4-IgG4m)	The treatment reduced MBP proliferation and IFN-γ secretion by MBP-specific cells with only mild side effects.	[170]
Phase II, randomized, clinical trial	65 RRMS	CTLA-4	Abatacept (CTLA-4 Ig fusion protein) or Placebo	No substantial differences were observed between the Abatacept and placebo groups in terms of the mean number of new Gd+ MRI lesions or other MRI and clinical parameters of disease activity.	[171]
Phase II, randomized, clinical trial	65 RRMS	CTLA-4	Abatacept (CTLA-4 Ig fusion protein) or Placebo	Abatacept treatment drastically lowered the relative frequencies of Treg and Tfh cells in circulating CD4^+^ T cells, as well as circulating plasmablasts. compared to a placebo. It suppressed their activity by downregulating activation markers, such as CD38 and ICOS genes, which are implicated in cell division and chromatin dynamics in these cells.	[172]
Case report	1 MS	CTLA-4	Abatacept (CTLA-4 Ig fusion protein)	After a two-year period, significant improvements were noted in MRI findings, demonstrating the absence of any definitive abnormal enhancing lesion in the brain parenchyma. These positive changes were accompanied by improvements in clinical symptoms.	[173]
Phase 1, open-label, clinical trial	12 RRMS	CD40L	Toralizumab/IDEC-131 (Humanized αCD40L)	The treatment resulted in an enhancement of the CD25+/CD3+ and CD25+/CD4+ ratios and a shift towards an anti-inflammatory cytokine response.The treatment resulted in an enhancement of the CD25+/CD3+ and CD25+/CD4+ ratios and a shift towards an anti-inflammatory cytokine response.	[174]

*Abbreviations*: *CD40L* Cluster of differentiation 40 ligand, *CTLA-4* Cytotoxic T-lymphocyte-associated protein 4, *Gd* Gadolinium, *ICOS* Inducible T-cell co-stimulator, *IFN-γ* Interferon-gamma, *MBP* Myelin basic protein, *MRI* Magnetic resonance imaging, *RRMS* Relapsing-remitting multiple sclerosis, *Tfh* T follicular helper, *Treg* Regulatory T cell

Collectively, despite some controversies surrounding the pathological role of CTLA-4 in the context of EAE and MS, several preclinical studies utilizing EAE mouse models have shown promising results. These findings have prompted several randomized clinical trials, which have reported favorable outcomes in terms of cellular-level and clinical measures, as well as improvements in MRI findings. However, to fully evaluate the efficacy and safety of targeting CTLA-4 as a therapeutic intervention for MS, further large-scale clinical trials are necessary. In addition, optimizing dosing and administration, as well as exploring potential synergies with other therapeutic strategies, may further enhance the therapeutic potential of targeting CTLA-4 in MS.

## Stimulatory immune checkpoints in MS

### CD40

The cluster of differentiation 40 (CD40) alongside its ligand, CD40L, are transmembrane proteins from the TNF receptor superfamily. They are crucial components in the induction and maintenance of inflammatory response [175]. The interaction of CD40/CD40L between T cells and APCs induces bi-directional signaling, including forward and reverse signals, that leads to activation and differentiation of T cells /B cells and APCs, respectively. This dyad also regulates Th1 differentiation, cytotoxic T lymphocyte (CTL) activation, and memory CTL maintenance, establishing an amplification loop in the immune response [176]. Having said that, scientists have lately discovered its significance in neurological complications. CD40-CD40L-mediated neuroinflammation enhances BBB permeability, exacerbates edema, neuronal and glial cell injury, and accelerates the development of occlusive microthrombi [177].

An association has been discovered between certain CD40 SNPs and MS. In this way, two SNPs in the CD40 gene, rs6074022, and rs1883832, were shown to have a strong correlation with vitamin D deficiency at disease onset in a survey of 218 Jordanian MS patients [107]. Nonetheless, they were unable to show a substantial association between these SNPs and MS [178]. Further research revealed that the rs1883832C>T SNP could significantly downregulate CD40 mRNA expression, with individuals carrying CT and TT genotypes having lower levels of CD40 mRNA than those with CC [179]. The combined impact of the CD40 and CD40L polymorphisms on MS susceptibility and progression in a Polish population was also evaluated. It was discovered that individuals with the TT and CT genotypes at the CD40 rs1883832 loci had a higher risk of developing MS than those with the CC genotype, with the risk being greater in TT individuals. Individuals with the CC genotype, on the other hand, had an average MS diagnosis 2 years earlier than those with the TT and CT genotypes and were more likely to acquire a secondary progressive course of the disease [180]. Moreover, according to genome-wide association screens (GWAS), SNP rs6074022 had the highest association with MS and was shown to have the strongest correlation with CD40 expression among the 30 SNPs genotyped from the CD40 genomic region [181]. In this regard, Sokolova et al. demonstrated in a meta-analysis that the association between rs1883832 and MS is driven by linkage disequilibrium (LD), whereas rs6074022 is either a marker in greater LD with the functional variation or the functional variant itself [182]. Another study found that rs4810485*T is associated with reduced cell-surface expression of CD40 in all B cell subtypes, lower total CD40 expression, and lower IL-10 levels, demonstrating a robust genotype-phenotype correlation for CD40 across B cell differentiation [183]. Based on genetic polymorphisms of the CD40 locus, researchers discovered a decline in CD40 expression on the surface of B cells and monocytes in remitting MS patients, independent of genotype, a tendency that was amplified in patients with the CT SNP [184].

Reverse transcription polymerase chain reaction (RT-PCR) analysis of PBMCs from MS patients revealed considerable systemic upregulation of CD40 and CD40L mRNAs as compared to healthy controls [185], but not at the protein level, with no difference across clinical MS subgroups or disease stage [186]. Additionally, compared to healthy controls, MS patients exhibited a considerably higher number of CD40^+^CD4^+^ T cells (Th40), which were mostly memory phenotypic cells acting as an intermediary between Th1 and Th17 phenotypes and producing IL-17 and IFN-γ with a considerable portion of them simultaneously [187]. Also, when monocytes were stimulated with lipopolysaccharide (LPS) in vitro, it was shown that MS patients' monocytes had considerably greater levels of CD40 expression than healthy controls, suggesting that MS patients' monocytes are potentially more effective in co-stimulating T-cell activation than healthy controls [188]. In contrast to progressive MS patients, an *ex vivo* study of B cells from RRMS patients also revealed a substantial upregulation of CD40 [189]. In another study, however, B cells from RRMS and SPMS patients exhibited CD40 expression comparable to healthy donors [190]. According to mouse model studies, younger mice are protected from CNS autoimmune disease due to lower levels of MHC class II and CD40 expression on APCs, which prevents them from developing encephalitogenic Th1- and Th17 effector T cells [191]. Similarly, latent infection with γ-herpesvirus 68 (γHV-68), which is equivalent to human EBV, enhances CD40 expression on APCs, which results in a decline in Treg cells frequency and an increase in CD8^+^ T cell activation and CNS infiltration, promoting susceptibility to EAE [192].

Disruption of BBB in MS subjects allows the attraction of inflammatory cells into the brain, which considerably deranges myelinated axons. Inflammatory lesions caused by MS were evidenced to be followed by disturbance of BBB, mediated by CD40 [193, 194]. Further pathological studies of demyelinated MS plaques revealed the presence of microglia/macrophages and T lymphocytes expressing CD40 and CD40L, respectively [195]. Once T cells expressing CD40L enter CNS and activate CD40 on microglia, cytokines, nitric oxide, and matrix metalloproteinases are released, resulting in demyelination [193]. B cells expressing CD40 have also been detected inside the inflammatory lesions of MS patients, suggesting that CD40-mediated antibody production by B cells may contribute to MS pathogenesis [196].

Mechanistically, the immunological cascade in MS commences with CD4^+^ T cell activation, which leads to increased CD40L expression on T cells and facilitates co-stimulation of APCs via CD40, culminating in the production of IL-12 and IL-18, which stimulates T cells to produce IFN-γ [197]. CD40 signals also promote B10 pro cell IL-10 competence, with IL-21 driving B10 cell proliferation and effector cell formation, resulting in local IL-10 synthesis that dampens antigen-specific T cell responses throughout cognate interactions without triggering immunosuppression [198]. IL-10 production by B-cells during MS relapses is modulated through the interplay between TLR4 and CD40 signaling, implying that CD40 potentially contributes to recovery from MS relapse if signaling takes place concurrently with TLR4 [199]. Furthermore, it was demonstrated that DCs derived from both RRMS and SPMS patients have an upregulated expression profile of CD40 but induce different effects, with DCs from RRMS patients inducing higher levels of Th1 (IFN-γ, TNF-α) and Th2 (IL-4, IL-13) cytokines, whereas DCs from SPMS patients only induced a polarized Th1 response [200]. The physiologic effect of CD40 stimulation, on the other hand, is to increase brain-derived neurotrophic factor (BDNF) release and exhibit neuroprotective effects, which are lacking in MS patients despite rising CD40 expression in their monocytes, leading to deviated immunity in MS and persistent CNS neuronal loss [201].

Several studies have been conducted to investigate the potential consequences of presently utilized DMTs in the regulation of CD40 in MS patients. In this way, it was demonstrated that IFN-β treatment in RRMS patients could significantly upregulate the expression of CD40 on monocytes compared to untreated patients, reinforcing monocyte responsiveness in the release of neuroprotective factor BDNF via CD40 stimulation [202]. Thus, CD40 expression can be used to assess clinical response in patients receiving IFN-β therapy at early time points, with a lack of enhanced expression predicting therapeutic failure [203, 204]. Conversely, IFN-β was shown to significantly suppress excessive CD40L expression in T cells of MS patients compared to their untreated counterparts, resulting in lower levels of CD3^+^CD40L^+^ and CD4^+^CD40L^+^ T cells in the treated patients [205] Interestingly, DCs from RRMS patients treated with GA displayed considerably decreased CD40 expression compared to their untreated counterparts, which is associated with relapse risk [206].

Blocking CD40 is believed to be effective in relieving EAE, making it an intriguing intervention [74, 207–210]. In this respect, anti-CD40 Ab or 8-oxo-dG in the EAE model were shown to positively regulate IL-10- and IL-35-producing Foxp3^+^ Treg cells that suppress activation and migration of mast cells in the brain via suppressing Ca^2+^ downstream signaling cascades and reducing the expression of CCL2/CCR2 and adhesion molecules, respectively, resulting in suppressed release of inflammatory cytokines. Anti-CD40-mediated enhanced Treg cell level also downregulates the Act1 in the mast cells activated by cytokines, notably IL-17, thus mitigating EAE severity [73]. Furthermore, the 6-amino-acid peptide sequence KGYY_6_ was a CD40-targeting peptide that Vaitaitis et al. investigated using an EAE mouse model. It shares 83% homology with mice and contains three amino acids required for interaction with CD40 [211–213]. It was discovered that KGYY_6_ binds to Th40 and memory T cells, upregulating the expression of CD69 and IL-10 in the CD4^+^ T cell compartment. This ultimately impedes the progression of the disease and alleviates symptoms, both when pretreated and when treated after the first symptoms were noticed. However, pretreatment with boosters had a greater impact than treatment administered individually [75]. Likewise, Toralizumab is a humanized anti-CD40L mAb with murine-determining regions consisting of human gamma 1 heavy chains and human kappa light chains. It only binds to human CD40L on T cells, inhibiting CD40 signaling [174]. Fadul et al. conducted a phase 1 clinical trial to evaluate the effect of Toralizumab/IDEC-131 on CD40 blockade. They enrolled 12 patients between the ages of 18 and 54 who had clinically definite diagnoses of RRMS, clinical relapse in the preceding year, and an EDSS of 0-3.5. The mAb was administered intravenously over one hour, followed by a two-hour monitoring period. The subjects were given four doses every other week. This mAb's circulation half-time was approximately 12-13 days [174]. The primary cohort received a dose of 1 mg/kg, and if no severe toxicity was observed, the dose was raised in each successive cohort to 5, 10, and 15 mg/kg. The therapy was well-tolerated and deemed safe, with only mild to moderate adverse effects reported during the study. An important finding was that none of the treated subjects experienced thromboembolic events, and the administration of this mAb did not exacerbate the disease [174]. One key concern in blocking CD40L is the increased risk of infection; however, earlier trials using this antibody to treat other autoimmune diseases found no specific increased risk of infection with a clear relation to the drug [214–216]. According to Fadul et al., only two patients developed an infection and both were in the 15 mg/kg treatment group, with one developing herpes zoster that was treated without sequelae [174].

Given the well-established pathological role of the dysregulated CD40/CD40L pathway in the development of neuroinflammation, the blocking of this pathway has garnered significant attention in preclinical and clinical studies on MS. Promising results have been reported, however, further research is still imperative to fully elucidate the role of this pathway in the development of the disease, address the side effects that have been observed in clinical trials, enhance its therapeutic efficacy, and perform large-scale studies to evaluate its potential as a therapeutic target for MS.

### CD137

CD137 (4-1BB), a tumor necrosis factor superfamily (TNFSF) receptor, has been extensively researched, particularly in cancer immunotherapy. It is found on activated T cells, but its ligand, CD137L, is expressed on APCs. CD137/CD137L signaling activation drives clonal proliferation and differentiation of T cells into IFN-γ-producing CD8^+^ T cells, and CD137 on T cells interacts with CD137L on APCs, prompting monocytes and macrophages differentiation to secrete proinflammatory cytokines [217].

CD137 mRNA level in blood CD4^+^CD25^+^ Tregs was found to be significantly lower in MS patients compared to patients with other neurological diseases and healthy controls [218, 219]. Likewise, CD137^+^ cells were detected in both the brain parenchyma and the brain blood vessels of post-mortem MS brain samples; active demyelinating lesions had the highest frequency of CD137^+^ cells, and specifically, CD137^+^ B lymphocytes were found accumulating in leptomeningeal infiltrates. Alongside enhanced cellular proliferation, CD137 signaling into B cells stimulates early TNF release and enhances IL-6 production, which are implicated in MS pathogenesis [220]. However, studies have not discovered CD3^+^ CD137^+^ T cells in MS lesions other than those with diffuse white matter abnormalities, nor in active, mixed active/inactive, or inactive lesions [221].

Research has shown that CD137 signaling is implicated in microglia activation, as CD137L-deficient mice exhibited much lower microglia activation in EAE [222]. Microglial activation triggers the phagocytosis of axonal myelin sheaths and the oligodendrocytes death, antigen presentation to T cells, and the production of proinflammatory cytokines in active lesions, which are the major pathogenic mechanisms underlying EAE and MS [223]. Consistently, in vitro*,* co-culture demonstrated that CD137L-activated microglia provoked apoptosis in oligodendrocytes via the generation of reactive oxygen species (ROS) [222]. Additionally, the identification of CD137-expressing apoptotic human Purkinje neurons in close contact with activated microglia suggested that activation of microglia via CD137L/CD137 signaling may potentially play a critical role in neuronal loss during MS [222].

**Fig. 1:**
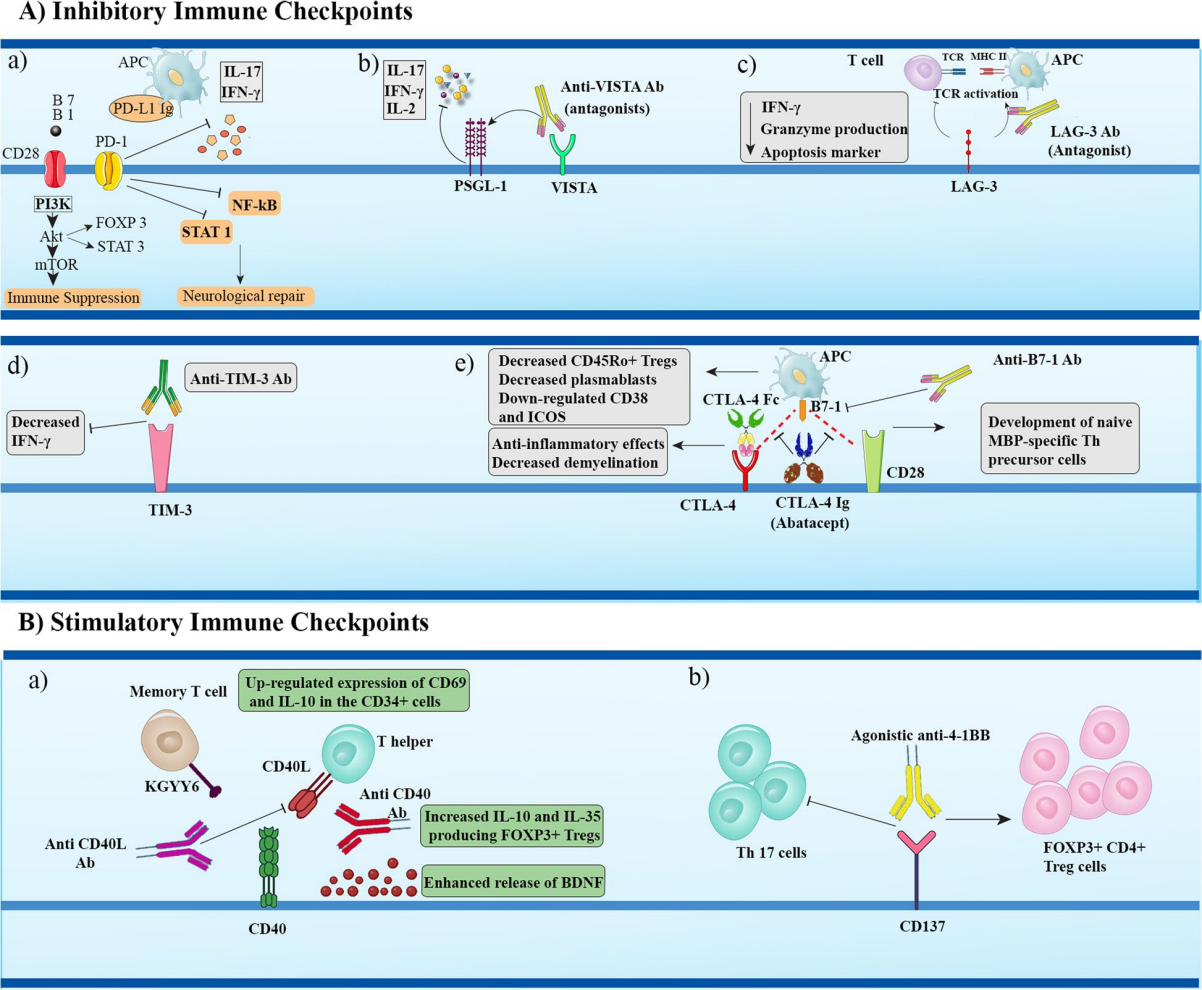
Summary of immune checkpoint-based immunotherapies for MS/EAE. *Abbreviations*: Ab: Antibody; APC: Antigen presenting cell; CTLA-4: Cytotoxic T-lymphocyte-associated protein 4; FOXP3: Forkhead Box P3; IFN-γ: Interferon gamma; IL-17: Interleukin 17; LAG-3: Lymphocyte activation gene 3; MBP: Myelin basic protein; MHC II: Major histocompatibility complex class II; mTOR: Mechanistic target of rapamycin; NF-κB: Nuclear factor kappa B; PD-1: Programmed cell death protein 1; PI3K: Phosphoinositide 3-kinase PSGL-1: P-selectin glycoprotein ligand 1; STAT3: Signal transducer and activator of transcription 3; TCR: T-cell receptor; Th: T helper; TIM-3: T-cell immunoglobulin and mucin-containing protein-3; VISTA: V-domain Ig suppressor of T cell activation

Given the above considerations, CD137-enhancing therapies have been investigated for the treatment of EAE and MS [224]. In this way, treatment of EAE mice with agonistic anti-4-1BB dramatically reduced disease incidence and ameliorate the disease severity; however, adoptive transfer of T cells obtained from mice treated with MOG_35-55_ was unable to avert EAE even after boosting their activity with anti-4-1BB, suggesting that anti-4-1BB therapy of EAE is only effective when administered during the disease induction phase [76]. Mechanistically, 4-1BB stimulation provokes the expansion of IFN-γ-releasing CD8^+^ T, resulting in indoleamine 2,3-dioxygenase (IDO)-dependent regulation of autoimmune responses. Anti-4-1BB Abs were also shown to suppress Th17 cells and related pathogenic IL-17 release while raising Foxp3^+^ CD4^+^ Treg cell frequency in an IFN-γ-independent manner, leading to the alleviation of EAE symptoms [77] (Fig. 1). These findings suggest that anti-4-1BB Abs have an immunoregulatory role in maintaining the equilibrium between Th17 and Treg cells. Despite these promising results, delivery of anti-4-1BB Abs to Gal-9 defective EAE mice failed to suppress the disease, revealing Gal-9 as a 4-1BB-associating protein and a pivotal regulator of anti-4-1BB immunotherapeutic activity. In this respect, by directly interacting with 4-1BB at a location distinct from that of antibodies and the 4-1BBL, Gal-9 was shown to enhance and stabilize 4-1BB aggregation, signaling, and activity in T cells, DCs, and NK cells [225]. In addition, researchers have investigated the therapeutic potential of CD137L suppression in EAE. In this way, CD137L knockout in EAE mice was confirmed to protect the mice from demyelination and EAE development by restricting encephalitogenic T cell activation and inflammatory cytokine release, as well as their trafficking into the CNS by downregulating vascular cell adhesion molecule-1 expression (VCAM-1) [78].

In summary, CD137/CD137L aberrations are implicated in EAE and MS pathogenesis. While preclinical studies have shown promise in using CD137-enhancing therapies to treat MS, further research is needed to fully translate these findings into clinical approaches for patients.

## Soluble immune checkpoints in MS

What’s more, there are a series of soluble ICPs alongside the ones located on the cell membrane, which are of utmost significance in immune regulation. sPD-1/sPD-L1 is mostly a result of proteolytic cleavage of the PD-1/PD-L1 bound to the membrane [226]. The mechanism by which sPD-1/sPD-L1 act in MS is not yet well understood. Studies found that sPD-1/sPD-L1 expression is upregulated in patients with autoimmune disease compared to the control group while a downregulation was observed after treatment, which suggests that sPD-1 can resist the inhibitory effect of PD-1 on T-cells, indirectly promoting immune responses [227–231]. As well, It was discovered that sCTLA-4 concentrations were lower in MS patients compared to controls and that sCTLA-4 did not correlate with the EDSS score in patients with MS and neuromyelitis optica [232]. sCD40L is another soluble protein that is mainly a product of activated platelet cleavage [233, 234]. Studies revealed that serum sCD40L was higher in MS subjects compared to control groups [235, 236]. Also, sCD40L was similarly increased in the CSF of MS patients in comparison with other neurological diseases [194]. The interesting finding is that serum concentration of sCD40L was decreased following treatment with natalizumab [237], GA [238], and IFN-β [239], which represents sCD40L as a biomarker for assessment of the efficacy of the therapies. Du and his team found out that the albumin index was positively correlated with serum sCD40L in MS, determining its role in BBB permeability [240]. The high serum concentration of sCD137, a product of differential splicing, was detected in autoimmune disease subjects including MS [241, 242]. An earlier study on MS subjects indicated that CD137 expression on CD4^+^CD25^+^ Tregs was decreased compared to CD4^+^CD25^−^ T cells [218]. A decrease in the expression of CD137 on the cell membrane may result in lower levels of sCD137 secretion, implying that enhancing CD137 signaling could have a beneficial effect on sCD137 production in autoimmune diseases.

Collectively, soluble ICPs have been recognized as an important factor in the pathogenesis of MS, but the current understanding of their precise pathological role in the disease is still preliminary. To fully appreciate their potential therapeutic opportunities, additional studies are needed.

## Conclusion

MS pathogenesis is essentially based on over-activated immunity targeting autoantigens, which is triggered and mediated by genetic and environmental factors; thus, any evidence of dysfunctionality in immunoregulatory mechanisms in the way to overstimulate immune responses while suppressing inhibitory mechanisms may provide a comprehensive view of the disease's etiopathogenesis and a rational therapeutic target to address. Among them, ICPs, which play a crucial immunoregulatory role in maintaining tolerance to self-antigens, have been shown to exhibit a variety of distinct SNPs and a dysregulated expression pattern in MS patients and EAE models. These differential expression patterns are not only confined to MS patients and healthy controls but also varied with clinical phenotype and disease course, establishing ICPs as prospective diagnostic and prognostic indicators for MS.

Multiple immunotherapeutics have been developed to target ICPs in MS and EAE. In this approach, immune cells such as T cells, B cells, and DCs have been engineered to overexpress ligands such as PD-L1 to prevent autoreactive immunity, with encouraging results in EAE models. Similarly, multiple Ig fusion proteins, including PD-L1 and CTLA-4 Ig fusion proteins, were developed to suppress co-stimulatory interactions, resulting in protection against MS/EAE and considerable disease amelioration. Furthermore, several methods, such as Ig-mediated targeting of stimulatory ICPs such as anti-CD40L Ab and anti-CD137 Ab, as well as CD40-targeting peptides such as KGYY6, have shown potential capacity in reducing the incidence and severity of EAE. Given these findings, multiple clinical trials with strong evidence of improved immunological profile, as well as radiological and clinical parameters in MS patients have been undertaken. One significant challenge is adjusting the amplitude of co-stimulation and co-inhibition to optimize efficacy while avoiding negative consequences, which has previously been addressed in cancer immunotherapy, where excess co-stimulation was coupled with autoimmune disease [243–245]. Thus, large-scale trials to optimize the clinical efficacy of ICP-based immunotherapies and establish an optimal combinational treatment with currently used therapies, as well as incorporating novel gene-modification strategies such as CRISPR/Cas9 to engineer immune cells with favorable ICP expression, are still needed.

## Data Availability

Not applicable.

## Abbreviations

APCs: Antigen-presenting cells
BBB: Blood-brain barrier
BDNF: Brain-derived neurotrophic factor
CD: Cluster of differentiation
CEACAM-1: Carcinoembryonic antigen-related cell adhesion molecule 1
CNS: Central nervous system
CSF: Cerebrospinal fluid
CTL: Cytotoxic T lymphocyte
CTLA-4: Cytotoxic T-lymphocyte antigen-4
DC: Dendritic cell
DMF: Dimethyl fumarate
DMTs: Disease-modifying therapies
EAE: Experimental autoimmune encephalomyelitis
EBV: Epstein-Barr virus
EDSS: Expanded Disability Status Scale
GA: Glatiramer acetate
Gal-9: Galectin-9
Gd+: Gadolinium-enhancing
GWAS: Genome-wide association screens
HMGB1: High mobility group protein B1
ICOS: Inducible costimulator
ICP: Immune checkpoint
IDO: Indoleamine 2,3-dioxygenase
IL: Interleukin
IP-10: Interferon-gamma-induced protein 10
IRF4: Interferon-regulatory factor 4
LAG-3: Lymphocyte-activation gene 3
LD: Linkage disequilibrium
LPS: Lipopolysaccharide
mAb: Monoclonal antibody
MBP: Myelin basic protein
MCP-1: Monocyte chemoattractant protein-1
MHC: Major histocompatibility complex
miRNA/miR: MicroRNA
MOG: Myelin oligodendrocyte glycoprotein
MS: Multiple sclerosis
NGS: Next-generation sequencing
NK: Natural killer
NMO: Neuromyelitis optica
NOD-2: Nucleotide-binding oligomerization domain-containing protein 2
PD-1: Programmed cell death 1
PPMS: Primary progressive MS
PSGL-1: P-selectin glycoprotein ligand 1
ROR-γt: Retinoic acid-related orphan receptor
ROS: Reactive oxygen species
RT-PCR: Reverse transcription polymerase chain reaction
RRMS: Relapsing-remitting MS
RUNX-1: Runt-related transcription factor 1
SNPs: Single-nucleotide polymorphisms
SPMS: Secondary progressive MS
TGF-β: Transforming growth factor beta
Th: T helper
TIM-3: T-cell immunoglobulin and mucin-containing protein-3
TLR4: Toll-like receptor-4
TNFSF: Tumor necrosis factor superfamily
VCAM-1: Vascular cell adhesion molecule-1
VISTA: V domain-containing Ig suppressor of T-cell activation
VSIG3: V-set and Ig domain-containing 3
